# Fatal Neonatal DOLK-CDG as a Rare Form of Syndromic Ichthyosis

**DOI:** 10.3389/fgene.2021.719624

**Published:** 2021-12-08

**Authors:** Katalin Komlosi, Olivier Claris, Sophie Collardeau-Frachon, Julia Kopp, Ingrid Hausser, Juliette Mazereeuw-Hautier, Nathalie Jonca, Andreas D. Zimmer, Damien Sanlaville, Judith Fischer

**Affiliations:** ^1^ Institute of Human Genetics, Medical Center, Faculty of Medicine, University of Freiburg, Freiburg, Germany; ^2^ Department of Neonatology, Hospices Civils de Lyon, Hôpital Femme Mère Enfant, Bron, France; ^3^ Claude Bernard University, Lyon, France; ^4^ Hospices Civils de Lyon, Hôpital Femme Mère Enfant, Institut de Pathologie, Bron, France; ^5^ Faculté de médecine Lyon Est, Claude Bernard University, Lyon, France; ^6^ Institute of Pathology, Heidelberg University Hospital, Heidelberg, Germany; ^7^ Dermatology Department, Reference Center for Rare Skin Diseases, CHU Larrey, Université Paul Sabatier, Toulouse, France; ^8^ Infinity, CNRS, Inserm, UPS, Université Toulouse, Toulouse, France; ^9^ CHU Toulouse, Hôpital Purpan, Laboratoire de Biologie Cellulaire et Cytologie, Institut Fédératif de Biologie, Toulouse, France; ^10^ Hospices Civils de Lyon, Hôpital Femme Mère Enfant, Service de Génétique, Bron, France; ^11^ Institut Neuromyogène, Université de Lyon, Lyon, France

**Keywords:** DOLK, congenital disorders of glycosylation, whole exome sequencing, Mendelian disorders of cornification, syndromic ichthyosis

## Abstract

Neonatal collodion baby or ichthyosis can pose a diagnostic challenge, and in many cases, only additional organ involvement or the course of the disease will help differentiate between non-syndromic and syndromic forms. Skin abnormalities are described in about 20% of the congenital disorders of glycosylation (CDG). Among those, some rare CDG forms constitute a special group among the syndromic ichthyoses and can initially misdirect the diagnosis towards non-syndromic genodermatosis. DOLK-CDG is such a rare subtype, resulting from a defect in dolichol kinase, in which the congenital skin phenotype (often ichthyosis) is later associated with variable extracutaneous features such as dilatative cardiomyopathy, epilepsy, microcephaly, visual impairment, and hypoglycemia and may lead to a fatal course. We report two neonatal cases of lethal ichthyosis from the same family, with distal digital constrictions and a progressive course leading to multi-organ failure and death. Postmortem trio whole-exome sequencing revealed the compound heterozygous variants NM_014908.3: c.1342G>A, p.(Gly448Arg) and NM_014908.3: c.1558A>G, p.(Thr520Ala) in the *DOLK* gene in the first affected child, which were confirmed in the affected sibling. Reduced staining with anti-α-Dystroglycan antibody was observed in frozen heart tissue of the second child as an expression of reduced O-mannosylation due to the dolichol kinase deficiency. In addition to the detailed dermatopathological changes, both cases presented hepatic and extrahepatic hemosiderosis on histological examination. Our patients represent an early and fatal form of DOLK-CDG with a striking presentation at birth resembling severe collodion baby. Both cases emphasize the phenotypic variability of glycosylation disorders and the importance to broaden the differential diagnosis of ichthyosis and to actively search for organ involvement in neonates with ichthyosis.

## Introduction

Pediatric disorders leading to perinatal death still present a challenge for genetic counseling when rapid whole exome/whole genome sequencing (WES/WGS) in the neonatal setting is not available. Affected children often die before a genetic diagnosis can be established and phenotypic descriptions are often insufficient. Complete and rigorous autopsy with multiple tissue sampling of affected fetuses or children is of great importance for detecting all anomalies that can indicate a potential diagnosis, and providing frozen tissue for genetic and functional analysis. A definitive diagnosis always requires a multidisciplinary approach including clinical data, autopsy findings, and genetic results, especially for correct interpretation of the pathogenicity of the identified DNA variants. A confirmed genetic defect is the requirement for targeted carrier testing in parents and prenatal or preimplantation genetic diagnosis in further pregnancies.

Inherited ichthyoses are classified as Mendelian disorders of cornification (MEDOC), which are further defined on the basis of clinical and genetic features and can be divided into non-syndromic and syndromic forms. To date, mutations in more than 50 genes are known to result in various types of ichthyoses (Fischer and Bourrat, 2020). The syndromic ichthyoses are generally very rare and are classified based on the mode of inheritance, and can be further subdivided according to the predominant symptoms (Oji et al., 2010; Fischer and Bourrat, 2020).

Rare forms of congenital disorders of glycosylation (CDG) are among the inborn errors of metabolism presenting at birth with ichthyosis. CDG are due to deficient glycosylation of proteins and lipids and are characterized by multi-organ symptoms with a wide range of clinical severity, from mild symptoms to severe multisystem dysfunction, and even a fatal course (Francisco et al., 2019). The spectrum of clinical manifestations comprises psychomotor delay and intellectual disability, muscle hypotonia, seizures, endocrine and coagulation abnormalities, ophthalmologic anomalies, failure to thrive, and variable dysmorphic features. Skin abnormalities are described in only about 20% of the different CDG forms (Rymen et al., 2012; Haijes et al., 2020; Komlosi et al., 2020). Generally, the skin manifestations represent only a single feature within a much broader phenotype and include orange peel skin, ichthyosis, increased skin laxity, hypo/hyperpigmentation, tumoral calcinosis, aplasia cutis congenita, hypohidrosis, hyperthermia, lipodystrophy, and psoriasis (Rymen et al., 2012; Kouwenberg et al., 2014; Alsubhi et al., 2017; Van Damme et al., 2017; Haijes et al., 2020; Komlosi et al., 2020). CDG linked to ichthyosis or ichthyosiform dry skin with variable neurologic and multi-organ involvement are due to deficiencies within the dolichol (DOLK-, SRD5A3-CDG) and the GPI (glycosylphosphatidylinositol) anchor biosynthesis pathway (PIGL-CDG, MPDU1-CDG) (Schenk et al., 2001; Kranz et al., 2007; Kahrizi et al., 2011; Lefeber et al., 2011; Kapusta et al., 2013; Jaeken et al., 2014; Rymen and Jaeken, 2014; Rush et al., 2017; Thiel et al., 2018; Hall et al., 2020; Ng et al., 2021). Recently, mild manifestations were also observed in a patient with COG5-CDG (Rymen et al., 2012). We recently described a family with two affected children with COG6-CDG in whom very dry, tight, and rigid skin with hyperkeratosis and scaling was the prominent skin feature at birth (Komlosi et al., 2020).

Construction of the complex N-linked glycan chains on proteins starts with production of the lipid carrier dolichyl phosphate, which serves to anchor the nascent oligosaccharide to the endoplasmic reticulum. It is known that dolichol and dolichyl phosphate metabolites are expressed ubiquitously in humans and are localized to the endoplasmic reticulum but also present in other organelles such as the Golgi apparatus, mitochondria, and lysosomes. In addition to their critical role in glycan synthesis, dolichols have a number of other proposed functions including modulating physiochemical properties of lipid bilayers and shielding of cellular lipids from oxidative damage (Cantagrel et al., 2010; Buczkowska et al., 2015). Dolichol kinase deficiency (DOLK-CDG, OMIM #610768) is an autosomal recessive disorder that results from deficiency of the enzyme responsible for the terminal step in the synthesis of dolichol phosphate. The range of symptoms and age at presentation are highly variable in the patients described so far (Kranz et al., 2007; Lefeber et al., 2011; Kapusta et al., 2013; Rush et al., 2017; Hall et al., 2020). The phenotypic spectrum encompasses three major forms with (1) neurological abnormalities with hypotonia, microcephaly, seizures, and visual impairment with or without ichthyosis; (2) isolated cardiomyopathy; and (3) multiorgan involvement, which can also include ichthyosis (Kranz et al., 2007; Lefeber et al., 2011; Helander et al., 2013; Kapusta et al., 2013; Lieu et al., 2013; Rush et al., 2017; Hall et al., 2020; Paprocka et al., 2021). A few cases with biallelic pathogenic variants in *DOLK* (OMIM *610746) with severe multi-organ involvement encompassing profound muscular hypotonia, ichthyosiform skin, nystagmus, epilepsy, and pulmonary infections leading to death within the first months of life have also been described (Kranz et al., 2007; Lefeber et al., 2011; Lieu et al., 2013; Rush et al., 2017; Hall et al., 2020).

We present two children of a non-consanguineous French couple with compound heterozygous variants in the *DOLK* gene presenting with severe ichthyosis, distal digital constrictions, cardiomegaly, thrombocytopenia, diffuse coagulation defect, and progressive multi-organ failure, which resulted in death in the neonatal period. They represent two additional cases of the early and fatal form of DOLK-CDG with a striking presentation at birth resembling autosomal recessive congenital ichthyosis with progressive multi-organ failure.

## Materials and Methods

### Clinical Report

#### Patient 1

The study was conducted according to the guidelines of the Declaration of Helsinki, and approved by the Ethics Committee of the University of Freiburg (ethical code number: 436/17). The parents of the patients gave written informed consent to the publication of the clinical information and the pictures.

**FIGURE 1:**
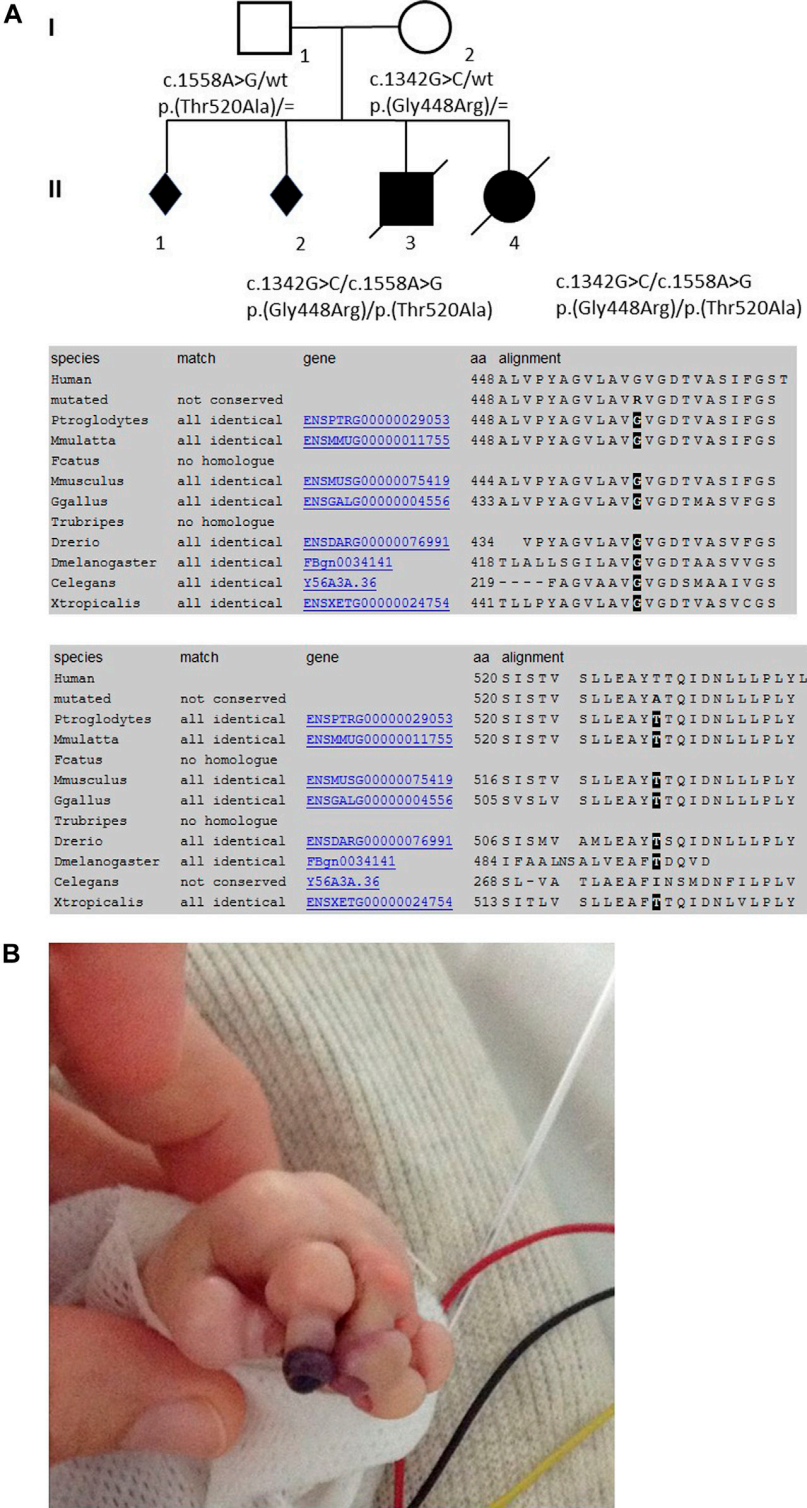
Pedigree of the family, conservation of the AAs among the diverse species (from MutationTaster, www.mutationtaster.org), and skin manifestations of the first child in the neonatal period.

Patient 1 was male and the first child of healthy French European parents (Figure 1A). The mother was a 30-year-old with a history of two spontaneous abortions. Pregnancy was complicated by oligohydramnios and intrauterine growth retardation. Prenatal ultrasound examinations did not show any malformation and the couple did not wish further prenatal genetic diagnosis. Pregnancy was marked by rupture of the amniotic membrane at 31 + 2 weeks of gestation and administration of antenatal corticosteroids. The infant was born prematurely at 35 + 1 weeks of gestation by vaginal delivery with a birth weight of 1,840 g (3rd percentile), length of 42.5 cm (3rd percentile), and occipitofrontal circumference (OFC) of 31.2 cm (10th percentile). Apgar scores were 10/10/10/10 at 1, 3, 5, and 10 min, respectively. Physical examination showed a congenital anomaly of all four extremities resembling amniotic bands with extremity edema and tendinous retractions at the level of the joints (Figure 1B), underdeveloped thenar, rigid fingers, signs of oligohydramnios with low-set ears, hypertelorism, broad forehead and flat nose, a narrow chest, and arthrogryposis (Supplementary Figure S1A). The most prominent feature was the very dry, nonelastic, and hard skin. Chest x-ray showed a narrow thorax and lung hypoplasia as a consequence of the oligohydramnios and cardiomegaly with right deviation of the heart. There was no sign of hepatosplenomegaly on physical examination, with no abdominal ultrasound performed. After an initial stable cardiac and respiratory state, the neonate developed progressive respiratory failure at 12 h of age and was transferred to a level III Neonatal Intensive Care Unit. He was difficult to ventilate adequately, and developed pneumomediastinum and pneumothorax requiring exsufflation. He was subsequently switched from conventional ventilation to high-frequency oscillatory ventilation. Sequential echocardiographs were performed showing severe persistent pulmonary hypertension, a large right-to-left shunting patent ductus arteriosus, and a mild but significant left ventricle hypokinesia with mild mitral and aortic insufficiency. Despite administration of dobutamine and norepinephrine, hypotension persisted and the child developed multi-organ failure after 48 h, with anuria, severe metabolic acidosis (pH 6.97), elevated lactate (15 mmol/L), thrombocytopenia (64 G/L), elevated transaminases, necrosis of the digits (Figure 1B), and a progressive respiratory failure. He died on postnatal day 3. The immediate cause of death was hemodynamic instability, ventricular fibrillation, and severe metabolic acidosis.

#### Patient 2

Patient 2 was female, born to the same parents 20 months after patient 1 (Figure 1A). The pregnancy was complicated by prenatal ultrasound findings of hyperechogenic intestines in gestational week 23. Conventional karyotyping and array CGH analysis from amniotic fluid was normal; *CFTR* screening revealed a heterozygous paternal mutation. After premature rupture of the amniotic membrane and chorioamnionitis, the infant was born prematurely at 32 + 4 weeks of gestation by vaginal delivery with a birth weight of 1,800 g (25th–50th percentile), a length of 44.5 cm (50th–75th percentile), and OFC of 29 cm (25th percentile). Apgar scores were 9/8/8/10 at 1, 3, 5, and 10 min, respectively. Physical exam showed collodion membrane with tight and hard skin with fissures, no ectropion or eclabion, and edema of the extremities (Supplementary Figures S1A,B). The fingers and toes were fixed in rigid volar/plantar flexion (Supplementary Figure S1B) and there was a blackish appearance of some fingers, raising the possibility of necrosis. Relieving incisions of the fingers (fingers II to V of both hands) were performed on day 4. There was no evidence of respiratory distress syndrome and the child was maintained on nasal CPAP (continuous positive airway pressure) until she developed apneic episodes and intubation was indicated. Although initially stable hemodynamically, on postnatal day 5, she developed several bradycardic episodes. There was no sign of hepatosplenomegaly and abdominal ultrasound was normal. Laboratory investigations revealed a diffuse coagulation defect (thrombocyte count: 201 G/L; total coagulation activity: 5.29xT, normal range: 0.8–1.2; Fibrinogen 1.8 g/L; Factor II: 9%, Factor V: 7%, Factor VII: 8%, Factor X: 16%) and plasma transfusion was administered. Ophthalmological examination showed extensive retinal hemorrhage of the right eye. On postnatal day 7, following episodes of crying and recurrent vomiting, the child developed a sudden cardiopulmonary arrest leading to death.

### Molecular Genetic Analyses

Since there was no confirmed genetic diagnosis during the neonatal period in either of the siblings, postmortem genetic analyses were performed. With the clinical suspicion of congenital syndromic ichthyosis, a multi-gene panel was initially performed from DNA extracted from a postmortem liver sample of the second child of the family. Mutation analysis of 66 genes associated with non-syndromic and syndromic ichthyosis (in-house designed HaloPlex Custom Kit, Agilent Technologies, Inc. Santa Clara, CA, United States) detected no pathogenic alterations.

For further analysis, trio whole-exome sequencing of the first child and the parents was performed using DNA extracted from a postmortem thymus sample and from peripheral blood of the healthy parents. Target enrichment of all coding genomic regions (exome) was performed with a Twist Human Core Exome Kit (Twist Bioscience) and sequencing was run on an Illumina platform (NextSeq500 System, San Diego, United States) with 150-bp paired-end reads. Candidate causative variants were validated by Sanger sequencing.

### Histopathological Analyses

Histopathological examination of the skin (H&E staining) was performed from the abdominal wall along the incision that was performed for internal examination in the first child (Figure 5A) and from the right inner thigh and the scalp of the second affected child (Figures 5B,C).

To assess glycogen storage in the liver and heart tissue of the first and second child, Periodic Acid–Schiff (PAS) and Periodic Acid–Schiff–diastase (PAS diastase) stainings were performed on FFPE tissues. For the detection of hemosiderosis, Perls staining was performed on FFPE of liver, spleen, pancreas, renal, thyroid, and thymus samples.

O-mannosylation was assessed by immunohistochemistry on frozen heart tissue sample of the second child with anti-α-Dystroglycan antibody (clone VIA4-1, Sigma-Aldrich Merck, Germany) directed against the O-mannosyl glycans of alpha-dystroglycan. As a control, a normal heart tissue sample from a healthy proband of the same age was used. To determine whether the antibody worked, a tissue sample from adult skeletal muscle was used.

## Results

### Molecular Genetic Results

Trio whole-exome sequencing revealed the compound heterozygous variants NM_014908.3:c.1342G>C, p.(Gly448Arg) and NM_014908.3:c.1558A>G, p.(Thr520Ala) in the *DOLK* gene in the affected first child. Sanger sequencing confirmed both variants in the DNA of the second child (Figure 1A). The variant NM_014908.3:c.1342G > C, p. (Gly448Arg), which is maternally derived, has not been described in the literature so far, nor is it listed in clinical databases (ClinVar or HGMD^®^ Professional 2020.3). In the population database gnomAD (the Genome Aggregation Database v2.1.1; http://gnomad.broadinstitute.org/), it is listed in heterozygous state in one individual (heterozygous carriers 1/251,402, allele frequency 0.0004%). Several *in silico* algorithms predict a deleterious effect of the variant and even an effect on splicing is predicted. Residue 448 is highly conserved among eukaryotes (Figure 1A). According to the ACMG guidelines (Richards et al., 2015) this variant was classified as likely pathogenic (class 4). The second variant NM_014908.3:c.1558A > G, p. (Thr520Ala), which is paternally derived, was already described (Retterer et al., 2016; Rush et al., 2017 and HGMD CM1618868) as pathogenic and results in a non-conservative amino acid substitution of a polar, hydrophilic threonine with a nonpolar, hydrophobic alanine residue. The Threonine-520 residue contributes to a highly conserved cytoplasmic domain (Rush et al., 2017, Figure 1A). Data from gnomAD suggest that this variant is rare, with an allele frequency of 0.004% (heterozygous carriers 9/251,490).

No further causative or possibly causative variants were identified in the trio analysis that would explain the striking phenotype in the siblings. Biallelic pathogenic variants in the *DOLK* gene are known to be responsible for DOLK-CDG (OMIM #610768).

### Results of the Macroscopic and Histopathological Analyses

**FIGURE 2:**
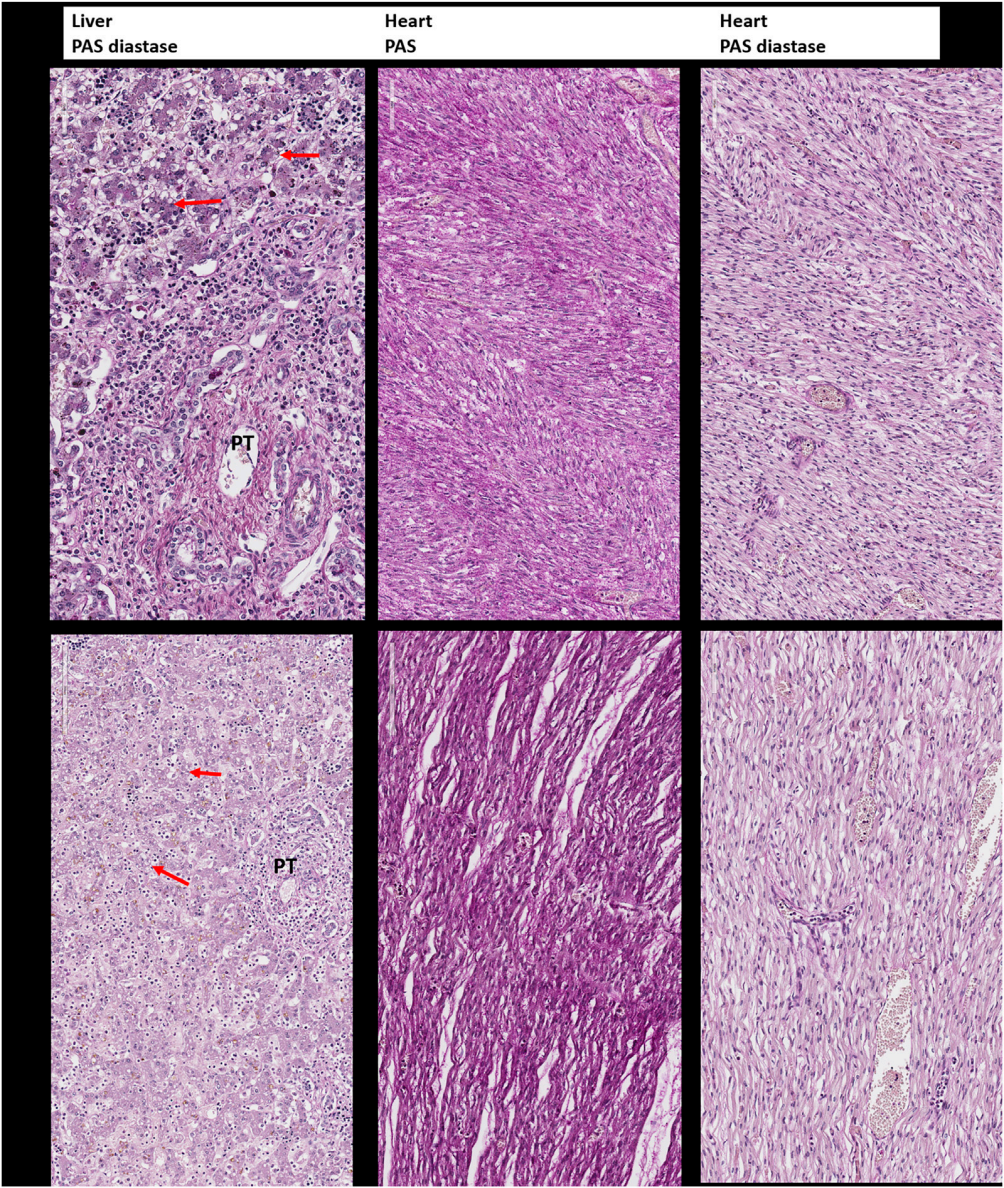
Periodic Acid–Schiff (PAS) and Periodic Acid–Schiff–diastase (PAS-D, PAS diastase) staining for the detection of glycogen. Upper panel: Liver and heart tissue of the first child: no evidence of glycogen storage and diffuse small vacuoles of steatosis in the hepatocytes (arrow). PT: portal tract. Lower panel: Liver and heart tissue of the second child with the same findings as in the brother.

**FIGURE 3:**
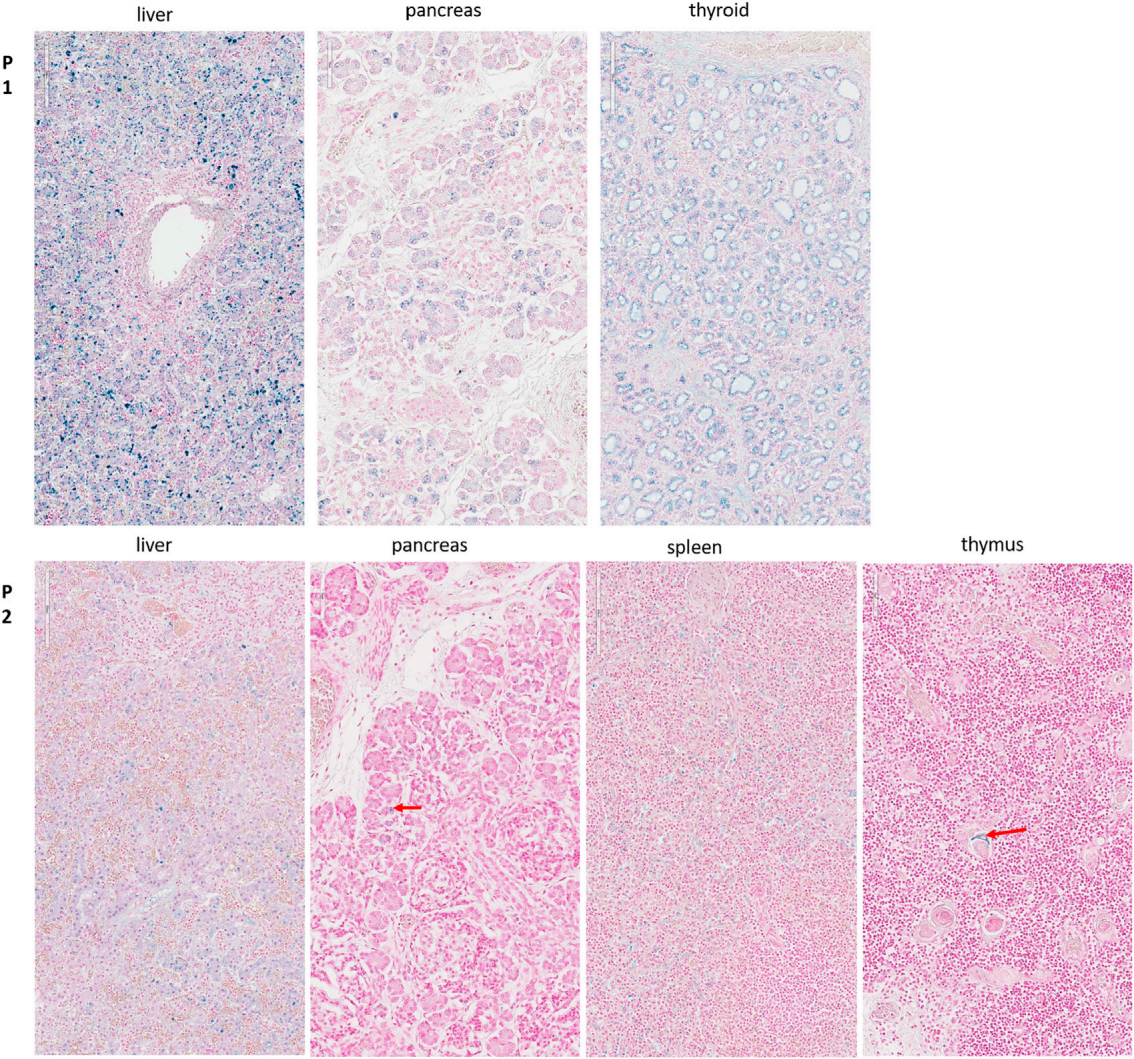
Perls staining for the detection of hemosiderosis. Upper panel (P1): Diffuse hemosiderosis in liver parenchyma, thyroid follicles, and pancreatic acini of the first child. Lower panel (P2): Liver: relatively diffuse hemosiderosis in liver parenchyma, less intense than in her brother; Pancreas: focal hemosiderosis in a few acini (arrow); Spleen: diffuse hemosiderosis in macrophages; Thymus: focal hemosiderosis in a few Hassal corpuscles (arrow).

In addition to the clinical anomalies previously described in Patient 1 (dry skin, pterygium, arthrogryposis of joints of all limbs, IUGR, cardiomegaly, and pulmonary hypoplasia, Supplementary Figure S1A), autopsy revealed a hypotrophic liver with diffuse steatosis (Figure 2, arrow) and hemosiderosis (Figure 3). There was no evidence of glycogen storage on PAS diastase staining in the liver (Figure 2). The heart weight was increased (18 g, normal value for age = 9.6 ± 2.4 g) without malformation, but ventricle wall measurement did not reveal hypertrophy and no steatosis or glycogen storage was seen on microscopic examination (Figure 2). Extrahepatic epithelial hemosiderosis was observed on Perls stainings in pancreatic acini and thyroid follicles (Figure 3, lower panel).

**FIGURE 4:**
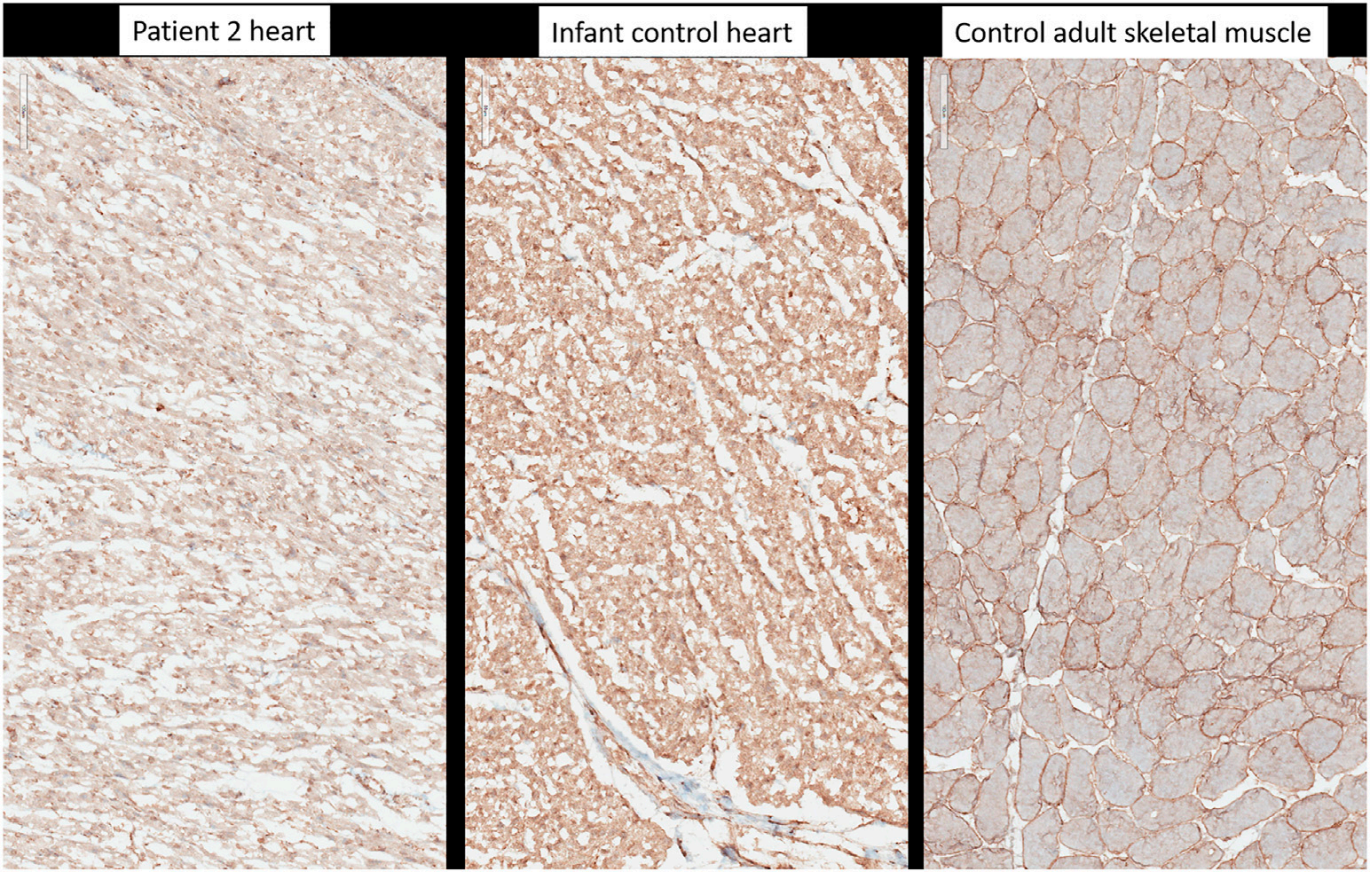
Immunohistochemistry with anti-alpha-dystroglycan (clone VIA4-1, Sigma-Aldrich-Merck, Germany). Heart tissue of patient 2, heart tissue of a healthy age-matched control, control from adult skeletal muscle.

On postmortem examination of the second child, there was no IUGR, but pterygium and arthrogryposis of all limbs and joints, as well as skin anomalies described at birth (Supplementary Figures S1B,C). In addition, autopsy highlighted cardiomegaly: the heart weighed 24 g (normal = 12.6 ± 2.9 g) with mildly increased right ventricular thickness (5 mm, normal = 3.2 ± 1 mm) without microscopic anomalies. No glycogen storage was seen on PAS and PAS diastase stainings (Figure 2). Immunohistochemistry with *α*-Dystroglycan antibody on frozen heart tissue showed a reduced expression compared to an age-matched control (Figure 4).

Liver weight in the second child was normal but diffuse steatosis (Figure 2) and hemosiderosis, less intense than in the brother, were present (Figure 3). Extrahepatic hemosiderosis was also seen on Perls staining in macrophages of spleen (diffuse hemosiderosis), while there was focal hemosiderosis in the thymus, in a few Hassal corpuscles (arrow), and in the pancreatic acini (Figure 3).

**FIGURE 5:**
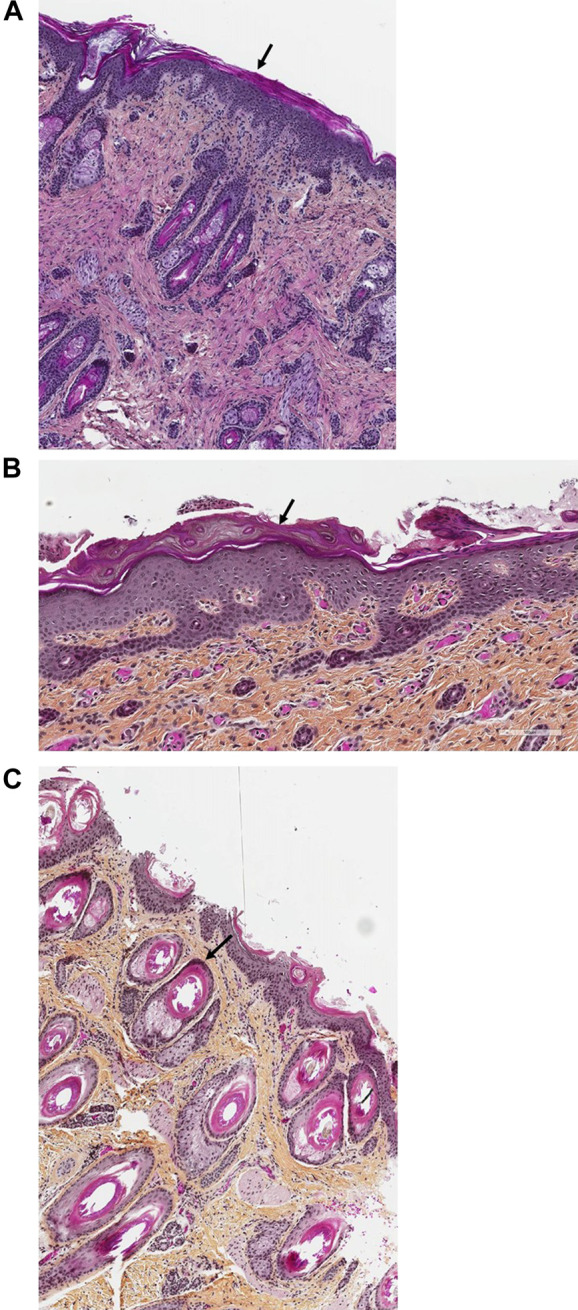
**(A–C)**. Skin histology of Patients 1 and 2. In the histopathological examination of the skin (H&E staining) from the abdominal wall along the incision that was performed for internal examination in the first child (Figure 5A, magnification ×40) and from the right inner thigh of the second affected child (Figure 5B, magnification ×100), the epidermis exhibited global compact orthohyperkeratosis with moderate to severe hyperplasia of the stratum corneum (arrows). The granular layer encompassed one cellular layer; there was acanthosis and no epidermal or sub-epidermal detachment (no bullae) and no inflammatory infiltrate with leucocytes or erythrocyte. Pronounced follicular plugging with onion scale-like keratin deposits were seen in the hair follicle orifices from the second child (Figure 5C, magnification ×100, arrow).

On histopathological examination of the skin from the abdominal wall (Figure 5A) of Patient 1, the epidermis exhibited global compact orthohyperkeratosis with moderate to severe hyperplasia of the stratum corneum (Figure 5A, arrow). The granular layer encompassed one cellular layer, and there was acanthosis and no epidermal or sub-epidermal detachment (no bullae) and no inflammatory infiltrate with leucocytes or erythrocyte (no signs of erythroderma). Histopathology from the right inner thigh of the second child was similar to those of her brother (Figure 5B); from the scalp, it showed pronounced follicular plugging with onion scale-like keratin deposits in hair follicle orifices (Figure 5C, arrow).

## Discussion

Here, we present two new cases of a lethal neonatal phenotype of DOLK-CDG whose severe ichthyosis at birth initially misdirected diagnostic efforts towards an autosomal recessive congenital ichthyosis. However, the combination of severe skin phenotype, distal digital constrictions, cardiomegaly, thrombocytopenia, coagulation defect, and multi-organ failure led to reconsideration and suspicion of an inborn error of metabolism. If CDG is suspected or considered early in the differential diagnosis of severely ill neonates, transferrin isoelectric focusing can be performed to screen for N-linked glycosylation defects as a rapid biochemical first-tier investigation. Due to the rapid fatal course in both of our patients, unfortunately, no isoelectric focusing of serum transferrin was performed. However, we attempted to show the effects of the compound heterozygous mutations of the dolichol kinase gene on reduced O-mannosylation in the second child. Dolichol-phosphate is converted to dolichol-phosphate-mannose, the monosaccharide donor for N-glycosylation inside the ER lumen and for O-mannosylation of alpha-dystroglycan. Immunohistochemistry with anti-α-Dystroglycan antibody directed against the O-mannosyl glycans of *α*-Dystroglycan on frozen heart tissue sample of the second child showed a reduced expression as an evidence of deficient protein mannosylation.

Dolichol kinase deficiency (DK) can present with a wide range and severity of symptoms (Kranz et al., 2007; Lefeber et al., 2011; Lieu et al., 2013; Rush et al., 2017; Hall et al., 2020). It can often be fatal, particularly when manifesting with cardiomyopathy. At the most severe end of the spectrum, two independent families with two and four affected children each, presenting with a lethal neonatal course including severe ichthyosis, digit skin constriction rings, dilated cardiomyopathy/cardiomegaly, hepatosplenomegaly, thrombocytopenia, hypoglycemia, and hypothyroidism, have been reported (Rush et al., 2017; Hall et al., 2020). Review of those most severely affected cases shows symptoms partly overlapping with those of our patients (Table 1). Besides presenting with striking congenital ichthyosis, both of our patients had a narrow chest and evidence of pulmonary hypoplasia on autopsy. In both children, evidence of hepatic and extrahepatic (spleen, thymus, thyroid, and pancreas) hemosiderosis was seen, which has only been described in a few cases of CDG so far (Agarwal et al., 2007; Léticée et al., 2010) and can lead to hematological, endocrinological, and immunological abnormalities. Endocrinopathy has already been described in other DOLK-CDG cases. Both of our patients had cardiomegaly; however, due to the rapid fatal course, they did not yet manifest signs of a dilatative cardiomyopathy. Deficient O-mannosylation of *α*-Dystroglycan in the heart tissue also points to a functional defect of the heart muscle in our second patient. Since altered glycosylation of certain coagulation factors can alter their plasma levels and thus influence the coagulation profile (Hansson and Stenflo, 2005), diffuse coagulopathies are often observed in CDG. The first child of the family showed necrosis of the digits already on the second day of his life probably due to thrombosis possibly caused by deficient glycosylation of clotting factors, e.g., Protein C and Protein S. The second child showed a severe diffuse coagulation defect with strongly decreased coagulation factor activities as an expression of a glycosylation defect and consequent intraretinal hemorrhage and required plasma transfusion.

**TABLE 1:** Clinical and laboratory comparison of eight DOLK-CDG cases with fatal neonatal or early infantile course.

	Patient 1	Patient 2	Rush et al., 2017 Patient 1	Rush et al., 2017 Patient 2	Hall et al., 2020, P1	Hall et al., 2020, P2	Hall et al., 2020, P3	Hall et al., 2020, P4
Gender	M	F	F	F	M	M	M	F
Age at presentation	Prenatal	Prenatal	At birth	Prenatal	Prenatal/at birth			
Age at death	2 days	7 days	8 days	2 months	9.5 h	24 h	26 h	6 days
Consanguinity	No	No	No	No	No			
DOLK variants	c.1342G > C, *p*. (Gly448Arg)/c.1558A > G, *p*. (Thr520Ala)	c.1342G > C, *p*. (Gly448Arg)/c.1558A > G, *p*. (Thr520Ala)	c.951C > A, *p*. (Tyr327*)/c.1558A > G, *p*. (Thr520Ala)	c.951C > A, *p*. (Tyr327*)/c.1558A > G, *p*. (Thr520Ala)	c.1262T > C, *p*. (Leu421Pro)/c1373G > T, *p*. (Gly458Val)			
Pregnancy history of the mother	Two spontaneous abortions	Two spontaneous abortions	Four spontaneous abortions	Four spontaneous abortions	Two early spontaneous abortions			
Pregnancy complications	Oligohydramnios and intrauterine growth retardation	Hyperechogenic intestines	Polyhydramnios	Polyhydramnios and apparent camptodactyly	Polyhydramnios, maternal hypoglycemia (2/4), reduced fetal activity (4/4)			
Gestational weeks at birth	35 + 1	32 + 4	32 + 3	33 + 1	39	40	38	36
Birth measurements	1,840 g (3rd *p*) 42.5 cm (3rd *p*) 31.2 cm (10th *p*)	1,800 g (25th–50th *p*) 44.5 cm (50th-75th *p*) 29 cm (25th *p*)	2,060 g (50th *p*) 48 cm (>97th *p*) NR	NR	2,580 g (3rd *p*) 45 cm (<3rd *p*) 33 cm (10th *p*)	2,720 g (5th *p*)	2,320 g (3rd *p*) 47 cm (10th *p*) 31 cm (3rd *p*)	2,381 g (25th *p*) 48 cm (50th *p*) 32 cm (25th *p*)
Weight								
Length								
OFC								
Facial dysmorphism	Low-set ears, large earlobe, hypertelorism, broad forehead, and flat nose	No, especially no ectropion or eclabion	Taut, shiny skin over the face, slightly downward slanting palpebral fissures	NR	Skin creases on forehead	NR	Relatively normal face	Normal face with forehead creases
Skin (macroscopic)	Collodion membrane with very dry, nonelastic skin	Collodion membrane with tight skin with fissures	Collodion membrane with taut, shiny skin over the entire body	Collodion membrane with taut, shiny skin	Generalized hyperkeratosis, thick skin, linear skin creases	NR	NR	Very thick and cracked soles
Skin histology	Diffuse global hyperkeratosis, compact orthokeratosis, hyperplasia of the stratum corneum, thin granular layer	Diffuse global hyperkeratosis, hyperplasia of the stratum corneum, dilated follicular ostiums, and pronounced follicular plugging	NR	Compact hyperkeratosis with focal parakeratosis, a normal granular layer, minimal dermal inflammation EM: lipid droplets in the stratum corneum	NR	NR	Hyperkeratosis of the skin	NR
Growth retardation	Yes	No	No	NR	3/4 yes			
Microcephaly	No	No	NR	NR	No			
Brain anomalies	NR	Periventricular hemorrhage	No	NR, autopsy declined	NR			
Cardiac pathology	Cardiomegaly	Cardiomegaly	Right ventricular hypertrophy, biventricular dilation, moderate-severe atrial dilation	Dilatative cardiomyopathy, first-degree heart block	Heart block, cardiomegaly			
Pulmonary manifestations	Pulmonary hypoplasia with focal alveolar bleeding	Thrombosis of the pulmonary arteries with calcification of the vascular walls	Lung development in the late canalicular or early saccular phase, delayed for age	NR	NR			
Gastrointestinal tract manifestations	Hypotrophic liver and steatosis hepatis	Episodic vomiting, hypotrophic liver, and steatosis hepatis	Hepatosplenomegaly with fibrosis, elevated transaminases	Elevated transaminases	NR	Hepatosplenomegaly	Hepatosplenomegaly	Splenomegaly
Digital and joint anomalies	Congenital anomaly of all four extremities resembling amniotic bands with extremity edema and tendinous retractions at the level of the joints, rigid fingers	Edema of the extremities, fingers, and toes fixed in rigid volar/plantar flexion, necrosis of the fingers	Necrosis of the distal phalanges of the hands and feet, sparing the thumbs and halluces fingers and toes fixed in rigid volar/plantar flexion	Milder digital constrictions, less severe digital amputation	Joint flexion, 10-digit circumferential skin constrictions with hypoplastic, absent, and/or clubbed nails	Additionally right knee contracture	Mild contractures of the large joints	Soft tissue lesion protruding from the dorsal surface of the distal phalanx of the second digit of the right hand
Hematologic abnormalities	Coagulation defect, bleeding, and necrosis	Diffuse coagulation defect	NR	Anemia thrombocytopenia	Anemia thrombocytopenia	NR	NR	Thrombocytopenia
Metabolic abnormalities	Severe metabolic acidosis, lactacidemia	Severe metabolic acidosis, lactacidemia	Hypothyroidism	Hypothyroidism	Hypoglycemia hypokalemia	NR	Hyperbilirubinemia	Hypoglycemia

It is interesting to note that the mothers in all three families reported with severe neonatal DOLK-CDG children had previous early miscarriages (Rush et al., 2017; Hall et al., 2020 and our family). In our family, no additional genetic cause was detected in the trio-exom sequencing, raising the possibility of a contribution of the *DOLK* mutation. Other prenatal manifestations in the fatal neonatal cases were IUGR (2/8), oligohydramnios (2/8), polyhydramnios (5/8), hyperechogenic intestines (in our case), and unexplained hypoglycemia of the mother during the pregnancy (2/8) (Rush et al., 2017; Hall et al., 2020 and Table 1).

Skin abnormalities have been described in about 20% of the different CDG forms (Rymen et al., 2012; Haijes et al., 2020; Komlosi et al., 2020). Recently, some rare forms have also been classified among the syndromic ichthyosis due to their characteristic skin manifestations (Fischer and Bourrat, 2020). Both siblings described here showed a striking congenital ichthyosis as previously described in this disease by Rush et al. (2017) and mentioned by Kranz et al. (2007) while in the four siblings with fatal neonatal DOLK-CDG recently described by Hall et al. (2020), less severe ichthyosis was seen. The pathomechanisms for the skin involvement in dolichol kinase deficiency are still not fully understood and there is only one previous report describing the detailed dermatopathological changes in this disease (Rush et al., 2017) while Hall et al. shows only the hyperkeratosis in one of the four siblings (Hall et al., 2020). Most theories explaining skin involvement are based on the assumption of the instability of key glycoproteins in skin. Since dolichol biosynthesis follows the sterol pathway up to the formation of farnesyl-PP, it has also been proposed that accumulation of toxic sterol precursors could contribute to the phenotype in patients with SRD5A3-and DOLK-CDG (Gründahl et al., 2012; Rymen and Jaeken, 2014). Since the diagnosis of a CDG disorder was established in our cases on postmortem samples, 3 years after the death of the second child in the family, no fresh tissue sample was available to assess the accumulation of a possible toxic metabolite precursor. Glycogen storage was not observed in the liver and heart tissue of both children on microscopic examination. One missense mutation found in our patients [*DOLK* NM_014908.3:c.1558A>G, p.(Thr520Ala)] was also found in a patient published by Rush et al. (2017), and the reported dermatopathological changes in this patient concurred with ours as well. Histopathology in our cases was typical for ichthyosis with global compact orthohyperkeratosis, thin granular layer, and follicular plugging (Figures 5A–C). Unfortunately, no skin specimen was available anymore for further ultrastructural analysis in our case. In the previous report by Denecke and Kranz (2009), electron microscopy of a skin biopsy sample revealed hyperkeratosis (Kranz et al., 2007), while Rush et al. showed evidence of abnormal lipid droplet accumulation in the stratum corneum and keratinocytes (Rush et al., 2017). With increasing awareness for rare metabolic disorders including CDG and with the expanding possibilities of rapid genetic diagnoses even in the neonatal setting, it will be very important to further elucidate the pathomechanism of skin and organ involvement in future confirmed DOLK-CDG cases.

## Conclusion

Our two cases presenting a detailed histopathology besides the clinical data expand the prenatal and postnatal phenotype of DOLK-CDG. They illustrate the broad differential diagnosis of neonatal syndromic ichthyosis and emphasize the importance to actively search for organ involvement in neonates with ichthyosis and to consider the congenital disorders of glycosylation among the possible diagnoses. Congenital ichthyosis with cardiac involvement and distal digital constrictions in combination with multi-organ failure and coagulation defects should prompt strong consideration of DOLK-CDG. In case of suspicion, a rapid first-tier screening using transferrin isoelectric focusing can be performed, followed by genetic analyses. Trio exome sequencing in children with severe disease course and early perinatal death can be valuable in informing the reproductive options of the affected families and allowing them access to prenatal and preimplantation genetic diagnosis.

## Data Availability

The original contributions presented in the study are included in the article/Supplementary Material. Further inquiries can be directed to the corresponding author.
