# Supplementary figures and images for: Fatal Neonatal DOLK-CDG as a Rare Form of Syndromic Ichthyosis
Source: Front Genet. 2021 Dec 8;12:719624. doi: 10.3389/fgene.2021.719624 (PMC8693085; doi:10.3389/fgene.2021.719624)

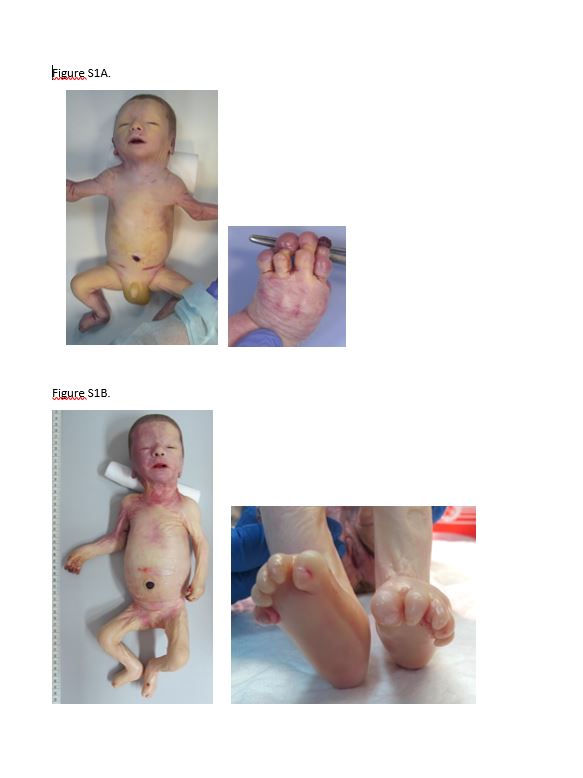

Supplement: Supplementary file 1 [file Image1.JPEG]
